# When Water Quality Crises Drive Change: A Comparative Analysis of the Policy Processes Behind Major Water Contamination Events

**DOI:** 10.1007/s12403-022-00505-0

**Published:** 2022-09-29

**Authors:** Nameerah Khan, Katrina J. Charles

**Affiliations:** School of Geography and the Environment, University of Oxford, Oxford, OX1 3QY UK

**Keywords:** Health, Multiple streams framework, Policy response, Policy windows, Water quality

## Abstract

The occurrence of major water contamination events across the world have been met with varying levels of policy responses. Arsenic—a priority water contaminant globally, occurring naturally in groundwater, causing adverse health effects—is widespread in Bangladesh. However, the policy response has been slow, and marked by ineffectiveness and a lack of accountability. We explore the delayed policy response to the arsenic crisis in Bangladesh through comparison with water contamination crises in other contexts, using the Multiple Streams Framework to compare policy processes. These included *Escherichia coli* O157:H7 and *Campylobacter* in Walkerton, Canada; lead and *Legionella* in Flint, Michigan, USA; and chromium-6 contamination in Hinkley, California, USA. We find that, while water contamination issues are solvable, a range of complex conditions have to be met in order to reach a successful solution. These include aspects of the temporal nature of the event and the outcomes, the social and political context, the extent of the public or media attention regarding the crisis, the politics of visibility, and accountability and blame. In particular, contaminants with chronic health outcomes, and longer periods of subclinical disease, lead to smaller policy windows with less effective policy changes. Emerging evidence on health threats from drinking water contamination raise the risk of new crises and the need for new approaches to deliver policy change.

## Introduction

In drinking water access, quality and quantity have been somewhat of a dichotomy (Nowicki et al. 2020), with quantity often prioritised. This has led to an ‘invisible water crisis’ of poor quality water (Damania et al. 2019). Throughout human history, water quality has been a constant and ongoing challenge—and yet certain crises have led to major changes in knowledge, policy, and regulatory structures. However, the pace and the drivers of change are often less clear: the cholera epidemic in 1850s London led John Snow to further our understanding of waterborne diseases (Damania et al. 2019), but London’s sanitary revolution was driven by political motivations and social priorities before the science was accepted (Charles et al. 2019). In recent years, there has been increasing recognition of the importance of drinking water quality and its impact on public health, and how such research can contribute to decision-making around the protection and management of water quality (Li and Wu 2019).

Policy responses to major water quality crises range between being proactive (i.e. prevention/mitigation), reactive (i.e. water treatment), and passive (i.e. inaction) (Damania et al. 2019). A major water quality issue that still remains unresolved to this day is the Bangladesh arsenic crisis; considered to be the largest-scale mass poisoning of a population (Yu et al. 2003). In order to gain a better understanding of why the response to arsenic has been so delayed, we examine water contamination crises in other contexts and compare why policy responses to such crises could differ.

We apply the Multiple Streams Framework (MSF) to examine the policy processes behind four water contamination events spanning different socio-political contexts, lengths of contamination, and types of contaminant. We explore policy responses in all four cases and examine a range of possible causes behind efficient and effective policy change versus delayed and ineffective policy change, encompassing the temporal nature of the disease, the visibility of the affected populations, the publicity received, and accountability mechanisms.

MSF was developed in 1984 by John Kingdon (Sabatier 2007) and has since been studied further, applied, and critiqued extensively (e.g. Cairney and Jones, 2016; Jones et al. 2016; Zahariadis, 2007). The framework posits that policies are created when three separate and independent streams—problems, policies, and politics—come together to create a policy window (Zahariadis 2007). When the window is open, policy entrepreneurs (i.e. major stakeholders in decision-making processes) can take action to initiate change. However, policy change is not usually successful if the political climate and public opinion are not favourable.

MSF is conducive to being used across a variety of contexts and has been applied as such (Jones et al. 2016)—both in terms of policy arenas and in terms of different countries, regions, and socioeconomic contexts. The ‘policy window’ aspect of this framework makes it an appropriate tool to assess the policy outcomes of sudden crisis or disaster situations.

## Methodology

This paper will frame the arsenic crisis within the global context of water quality and analyse the policy processes behind it, by comparing and analysing it against selected major water contamination events. This was done using the Multiple Streams Framework, so that the different levels of exploitation of the policy windows in each crisis can be compared and understood.

### Identification of Events

A varied set of water quality events were chosen purposively to compare to the Bangladesh arsenic crisis. Initially, phrases such as ‘water quality crisis’, ‘water quality and public health crisis’, ‘policy responses to drinking water crisis’, and ‘water quality crisis and policy processes’ were searched, and further literature was explored through snowballing. The criteria for choosing the events were as follows: contamination of drinking and domestic water supply; exposure to the contaminant as a result of human action (regardless of the contaminant itself being natural or anthropogenic); crisis resulted in risk to human health; and availability of adequate policy-related literature for comparison.

Based on these criteria, the events (and their starting years) which have been chosen for comparison are as follows:

- *Escherichia coli* O157:H7 and *Campylobacter* contamination of water supply in Walkerton, Ontario, Canada (2000);
- Lead and *Legionella* contamination of water supply in Flint, Michigan, USA (2014); and
- Chromium-6 contamination of private groundwater wells in Hinkley, California, USA (1987).

This is by no means meant to be an exhaustive list of water contamination events which meet the above criteria. It is rather a selective list of events which provide useful parallels for comparison. Lessons were taken from the use of the Multiple Streams Framework by Schwartz and McConnell (2009) in comparing the policy response to the water quality crisis in Walkerton with the banquet hall collapse in Jerusalem. However, this approach has not previously been used to compare water quality crises to each other.

### The Multiple Streams Framework

Selected water contamination events were assessed through the multiple streams approach to analyse what worked and what did not, in terms of policy response. Each event will be presented with an analysis of the three streams, the policy window, and the role that the policy entrepreneurs played within each event. The different aspects of the framework are described in brief detail below (Table 1).

**Table 1:** Components of MSF

MSF component	Description
Problem stream	Comprises problems or issues that can have negative effects—and are usually brought to public attention via indicators (e.g. disease incidence, mortality rates, etc.), focusing events (‘jarring and sudden’ (Jones et al. 2016, p. 15) occurrences related to a problem), and feedback (information or knowledge from similar events) (Zahariadis 2007). Within this stream, the problem load—i.e. how crowded the agenda is with various other issues—can determine if new problems receive attention (Zahariadis 2007; Jones et al. 2016)^a^
Politics stream	The political context within which a particular problem is situated, and the response a problem receives can be influenced by the national mood (public opinion on the issue), party ideologies (the views of the political parties within various government institutions), and balance of interests (views of interest/advocacy groups and other such stakeholders) (Zahariadis 2007; Jones et al. 2016)
Policy stream	A set of potential solutions to a specific problem. Not all solutions are applied, since this depends on the acceptability of the proposal, the technical feasibility, and the availability of necessary resources needed to implement it (Zahariadis 2007). The relevant policy community or network can also influence the uptake of specific solutions
Policy window	These are fleeting windows of opportunity which present themselves when the three otherwise independent streams join together, or are ‘coupled’ (Zahariadis 2007; Jones et al. 2016). These policy windows may open due to a problem arising (e.g. a major event) or due to changes in the politics stream (e.g. a new government), and they provide an opportunity for policy entrepreneurs to put their solutions forward
Policy entrepreneurs	These are actors who bring ‘the necessary dose of agency required to couple the streams and shape policy outputs’ and their success depends on the resources at their disposal, their access to relevant policymakers, and their strategies of manipulating the streams (Jones et al. 2016, p. 16)

^a^Since focusing events play an important role in this paper, to elaborate on the definition, ‘A focusing event is an event that is sudden; relatively uncommon; can be reasonably defined as harmful or revealing the possibility of potentially greater future harms; has harms that are concentrated in a particular geographical area or community of interest; and that is known to policy makers and the public simultaneously’ (Birkland 1998, p. 54)

A further note on policy entrepreneurs, Yanna Lambrinidou, an ethnographer who has done extensive work on lead in drinking water in the United States, mentions in an editorial:

In the end, technical interventions to “do good” can reinforce or disrupt the hegemony of the dominant social order—an order that privileges technical experts by granting them the right, authority, and even obligation to speak on behalf of affected communities about what these communities need and whether what they get is appropriate, sufficient, and effective. (Lambrinidou 2018, pp. 9–10)

In this paper we recognise that perhaps the very concept of a policy entrepreneur can be critiqued for perpetuating the discourse around specific people or entities having the agency to exploit windows of opportunity in policymaking, and thus limiting the space for collective action by affected communities to be taken into consideration by policymakers, media, and academics. Questions we seek to address in each case is whether policy entrepreneurs are amplifying citizens’ voices, or if policy entrepreneurs with the best intentions are helping ‘perpetuate the systematic erasure of community voices’ (Lambrinidou 2018, pp. 12–13), and if policymakers are less receptive to citizens’ voices.

### Analysis of the Literature

Academic literature on each of the events was studied by reviewing papers that describe the events overall. The next step was to review the literature around empirical evidence generated on the exposure to the contaminant and health outcomes related to these crises (where available). Further academic literature involving nuanced analyses or commentary on the events were examined, followed by grey literature, including reports (e.g. from an inquiry or taskforce) and government websites and resources related to the events. Aspects of the events were categorised into the components of the framework, i.e. problem stream, policy stream, politics stream, policy window, policy entrepreneur, and lastly the actual policy outputs and outcomes. Table 2 highlights the diversity of contexts within which the events took place, as well as some of the commonalities between them. Through the analysis, key themes were identified as they arose in the literature, such as acute versus chronic nature of the events; social and political context of each event; the politics of visibility (both of the outcome and the affected population); the level of public and media attention; and accountability and blame in each case.

**Table 2:** Characteristics of identified water contamination events

Indicators	Arsenic Various districts across country, Bangladesh (1993)^a^	*E. coli O157:H7, Campylobacter* Walkerton, Canada (2000)^b^	Lead, *Legionella* Flint, Michigan, USA (2014)^c^	Chromium-6 Hinkley, California, USA (1987)^d^
Scale & Population^e^	35 million people (estimated) exposed to arsenic above Bangladesh standard in 61 of 64 districts; *out of an overall 106.3 million population (1991 census)/124.4 million (2001 census)*	2300 cases, 7 deaths; *out of an overall 4851 population (2001 census)*	99,000 residents including approximately 9000 children exposed to lead; and 91 cases and 12 deaths from Legionnaire’s; *out of an overall 102,434 population (2010 census)*	650 people with various health concerns; *out of an overall 2026 population (1990 census)*
Geography	Mainly rural	Rural	Urban	Unincorporated community^f^
Source	Natural deposits in shallow groundwater	Pathogens from nearby farmland	Lead pipes, corrosive water from Flint River	Chromium-6 dumped in unlined ponds by electric company
Exposure	Switching from surface water to shallow groundwater	Inadequate chlorination; lack of compliance with testing procedures	Switching from Detroit Water (Lake Huron) to Flint River	Chromium-6 percolated into the groundwater
Pathways	Drinking/domestic water, crops	Drinking/domestic water	Drinking/domestic water	Drinking/domestic water
Democracy index^g^	5.99 (hybrid regime)	9.24 (full democracy)	7.92 (flawed democracy)	7.92 (flawed democracy)
GDP per capita (current US$)^h^	300.6 (1993; national)	24,271.0 (2000; national)	55,050.0 (2014; national) 28,330 (2014; Flint Metro Area)^i^	20,038.9 (1987; national)
Accessibility to healthcare services (i.e. physicians per 1000 people)^j^	0.2 (1991; national)	1.88 (2000; national) 1.80 (2000; Ontario)	2.75 (2014; national) 3.04 (2014; Michigan)	1.89 (1987; national) 2.17 (1987; California)

^a^More detail on Bangladesh arsenic crisis in next section; information in Table 2 from various sources (Smith et al. 2000; Kinniburgh and Smedley 2001; Human Rights Watch 2016); years stated are those of official recognition of each crisis

^b^More detail on Walkerton water crisis in next section; information in Table 2 from various sources (Salvadori et al. 2009; Schwartz and McConnell 2009)

^c^More detail on Flint water crisis in next section; information in Table 2 from various sources (Masten et al. 2016; Ranganathan 2016; Denchak 2018; Fasenfest 2019; CDC 2021b)

^d^More detail on Hinkley water crisis in next section; information in Table 2 from various sources (Banks 2003; Sutton 2010; California Water Boards 2021a)

^e^Census years closest to the year of recognition of each crisis (California Department of Health Services 2000; Bangladesh Bureau of Statistics 2011; Statistics Canada 2019; US Census Bureau 2021); except for Bangladesh where 2001 data are also shown since the exposure numbers were estimated then by Kinniburgh and Smedley (2001)

^f^An unincorporated area is not part of a municipality and is instead governed by the county or a nearby incorporated area (Cohen 2007); Hinkley is such a town located in the Mojave Desert

^g^As ranked and defined by the Economist Intelligence Unit Democracy Index in 2020 (The Economist Intelligence Unit 2021)

^h^All historic national data from World Bank DataBank (The World Bank 2022) unless otherwise stated. GDP specific to affected areas is difficult to pinpoint for Bangladesh as the contamination is spread across the country (more discussed on section “The Politics of Visibility”). Similarly, these data are not available for Walkerton (being a small town) and Hinkley (an unincorporated area) either. It is available for Flint, Michigan, however, likely since it is a larger metropolitan city

^i^Flint GDP data taken from Open Data Network (2022)

^j^Bangladesh data from World Bank DataBank (The World Bank 2022) from 1991—closest year with data available; data specific to affected population difficult to pinpoint as contamination is spread across the country (more discussed on section “Acute Versus Chronic Nature of Events”). Canada data from Canadian Institute for Health Information (2022). US data from National Center for Health Statistics (1990, 2018)

## Results

The results from the analysis of the literature show that each water contamination crisis was situated within a unique set of complex circumstances which led to the different levels of policy response. However, there are also many important parallels and similarities between them.

### Arsenic in the Groundwater, Bangladesh, 1980s/1990s

#### Problem Stream

In the 1970s–1980s, large-scale efforts were led by the government’s Department of Public Health Engineering (DPHE), along with UNICEF, to install shallow tubewells across much of the rural areas of the country. This was in an attempt to reduce diarrheal disease among the large number of people who depended on pathogen-containing surface water (Smith et al. 2000). This was a seemingly successful undertaking, because by the early 1990s, an estimated 2.5 million tubewells had been installed in rural areas, serving 95% of the population with ‘safe’ drinking water (Human Rights Watch 2016). However, in reality this water contained arsenic, and what was thought to be a successful campaign to provide safe water to citizens instead turned out to chronically expose them to a highly toxic substance.

The official recognition of the problem in 1993 (Atkins et al. 2007) provided a clear focusing event: there was feedback as a result of arsenic contamination in neighbouring West Bengal (i.e. knowledge from similar events) (Smith et al. 2000; Hanchett et al. 2014), and the subsequent diagnosis of arsenic-related skin lesions (Smith et al. 2000) provided a clear indicator of the problem.

#### Politics Stream

Government policies came at a delayed rate—about a decade after arsenic was first discovered—and the implementation of the policies has been slow as well (Atkins et al. 2007). According to Hanchett et al. (2014), the official recognition of the arsenic problem did not occur in Bangladesh until the 1990s, even though it was a recognised problem in the neighbouring Indian state of West Bengal since the 1980s—a delay which is not yet fully understood. Furthermore, Atkins et al. (2007) mention that the first patients from Bangladesh were identified in 1984, and Smith et al. (2000) state that by the mid-1980s to late 1980s, a doctor in West Bengal had identified several arsenicosis patients, including from Bangladesh. During this time he had ruled out other causes and determined tubewell water to be the source of arsenic ingestion (Smith et al. 2000). Additionally, according to Pearce (2001), it is possible the Bangladesh government was informed as early as 1985 regarding Bangladeshis who had been diagnosed in West Bengal.

Bangladesh is considered a ‘hybrid regime’ (Table 2)—with the current system of parliamentary democracy being established in 1991, after a period of dictatorship. Furthermore, there is an appearance of decentralisation but most power and decision-making lie with the national government, thus rendering local governments much less powerful (Fox and Menon 2008; Panday 2011). Much of the practical response regarding the arsenic crisis has been funded by foreign bilateral and multilateral donors, and implemented by NGOs (Atkins et al. 2007). Moreover, the policy response to the crisis has mainly involved a large-scale drive towards assessing the scale of arsenic contamination and increasing public awareness, an effort which largely ended in the mid-2000s (Hanchett et al. 2014). However, instances where there have indeed been efforts beyond just screening and awareness-raising have been fraught with issues of corruption. There has been misallocation of resources with regard to arsenic mitigation—i.e. interventions not being provided where most needed but where there are personal/political connections (Human Rights Watch 2016), often under the guise of community-based water management schemes (Rammelt et al. 2014). Similar information was previously reported by DPHE and the Japanese Government, who concluded that ‘The current situation analysis points to a tremendous gap between the number of installed safe water options and the areas with high arsenic contamination. Targeting high arsenic contamination areas is urgently needed’ (DPHE and JICA 2010, p. 62).

#### Policy Stream

The main policy solutions for the Bangladesh arsenic crisis were largely around screening water sources and mitigation (Hanchett et al. 2014). Mitigation solutions are mainly technological: involving switching to arsenic-free groundwater (i.e. from deeper aquifers), treated surface water, rainwater harvesting, and arsenic filtration (Caldwell et al. 2003; Hanchett et al. 2014). Other solutions included screening arsenicosis patients and large-scale awareness-raising programmes (Hanchett et al. 2002).

#### Policy Window

There was a window of opportunity following the realisation of arsenic contamination of groundwater in Bangladesh—but this window was not significant enough for prompt and effective action, or rather, there was a window but one which was not utilised effectively. Another issue to note is that the ‘problem load’ being faced by policymakers can also influence their uptake of information and the prioritisation of a specific issue. As mentioned earlier, Bangladesh is faced with multiple water quality and quantity hazards (such as salinity and groundwater depletion in addition to arsenic) (Shamsudduha et al. 2020), along with regular natural disasters, such as cyclones and floods (Karim and Mimura 2008). There is also much preoccupation with partisan politics (Atkins et al. 2007). Moreover, as Hanchett et al. (2014) points out, the World Bank invested much too large an amount of money too hastily—that is, without assessing best practices first. UNICEF, various NGOs and research institutions followed suit, resulting in a disparate range of short-term projects rather than coordinated long-term service delivery from the responsible government authorities (Atkins et al. 2007; Hanchett et al. 2014).

In 2002, a lawsuit was brought up against the British Geological Survey’s (BGS) parent organisation, the Natural Environment Research Council (NERC), in the UK courts for not testing for arsenic in a screening BGS conducted in 1992 (Ravenscroft et al. 2009). The *Sutradhar v. NERC* lawsuit was possibly a case of exploiting the wrong window of opportunity (Zahariadis 2007), since the judgement from the House of Lords states that BGS ‘owed no duty to the government or people of Bangladesh to test the water for anything’ (Sutradhar v. Natural Environment Research Council 2006, p. 10). This is because BGS was tasked with undertaking this particular hydrogeological survey as part of an agriculture project funded by the British government that sought to install 4000 deep tubewells for irrigation purposes, and had nothing to do with the drinking water project, which was funded by UNICEF that aimed to install over 4 million shallow tubewells (Sutradhar v. Natural Environment Research Council 2006).

#### Policy Entrepreneurs

It is difficult to pinpoint any single person, organisation, or entity as the policy entrepreneur behind the arsenic crisis. The closest possibilities are those responsible for the funding and implementation of large-scale screening which brought attention to the arsenic issue by presenting empirical data to highlight the magnitude of the issue, such as the World Bank and the BGS (through their later hydrogeological survey carried out between 1998 to 2001).^1^ The Bangladesh Environmental Lawyers Association would also count among the policy entrepreneurs, since they helped bring the NERC lawsuit to the UK courts through their international contacts (Atkins et al. 2006, 2007).

However, it should also be noted that the *Sutradhar vs NERC* lawsuit was also a case of ‘well-intentioned environmental lawyers’ taking a top-down approach to environmental justice (Atkins et al. 2006, p. 277). Binod Sutradhar may have had his name put forth as the plaintiff in the case, but how much agency did he or the other 511 clients on behalf of whom legal action was brought forth (Atkins et al. 2006) really have? He and the others were identified by the lawyers, and the case was taken up on their behalf (Atkins et al. 2006). In the case of the Bangladesh arsenic crisis, there has not been much in the way of community action or public outcry (Atkins et al. 2007), and those who have been affected have remained largely outside of the actions to respond to the crisis.

#### Policy Outputs and Outcomes

Atkins et al. (2007) constructed a timeline of events, highlighting some of the issues in the policy response to this crisis. Arsenic was found in Bangladesh’s groundwater in 1993, and in 1996 BGS was awarded a grant by the UK government to carry out a large-scale geological study to map out arsenic and other contaminants. The following year, the World Bank invested $44 million in the Bangladesh Arsenic Mitigation Water Supply Project (BAMWSP) where DPHE and UNICEF tested 51,000 wells across the country. The BGS survey was published in 2001, showing widespread arsenic in the shallow aquifers. A second phase of the BAMWSP was completed in 2003 with the help of various NGOs. In 2004, a new National Water Management Plan was approved with a subsidiary National Policy for Arsenic Mitigation, which covered issues, such as screening wells, identification and management of patients, mitigation, research, awareness-raising, and safe alternatives. However, as mentioned before, the actual implementation of mitigation measures has been lacking—ranging from nepotism in the placement of safer sources to a lack of diagnosis and management of those who are exposed (Human Rights Watch 2016). As Rammelt et al. (2014) state, ‘Drawing up a policy is one thing; implementation is another’ (p. 126).

A major intervention has been to paint tubewells below 50 µg/L arsenic green and those above this level red (Johnston and Sarker 2007), as a way to communicate the water quality to those vulnerable to exposure so they can make informed decisions when switching to other sources (Sultana 2011). However, switching sources is not so simple—since this comes with a range of issues, such as increased travel time to fetch water, sharing water sources, and navigating various socio-cultural complexities with regard to water access (Sultana 2009, 2011).

### *Escherichia coli* O157:H7 and *Campylobacter jejuni* in Walkerton, Canada, 2000

#### Problem Stream

Walkerton is a rural town in Ontario, Canada which faced an *E. coli* O157:H7 and *Campylobacter* outbreak in May 2000 (Salvadori et al. 2009). The source of the pathogens was identified as non-point source pollution (bacteria from manure) which washed into the town’s water supply from a nearby farm (Salvadori et al. 2009). This was further compounded by the karst hydrogeology of the area which makes it easy for contaminated surface water to reach groundwater sources (Prudham 2004). The contamination was attributed to operational failures and lack of chlorination, and this resulted in about 2300 cases of illness and seven deaths (Salvadori et al. 2009; Schwartz and McConnell 2009)*—*a clear focusing event. Moreover, it was later found that those who experienced acute gastroenteritis during the outbreak were at higher risk of developing hypertension, renal impairment, and cardiovascular disease (Clark et al. 2010). The water treatment plant operators did not have the proper training and were non-compliant with treatment, sampling, and monitoring procedures (Prudham 2004; Salvadori et al. 2009). However, the Ministry of Environment (MOE) was also held accountable for this outbreak since it was within their remit to ensure regulatory compliance: the inquiry which followed soon after (headed by Dennis O’Connor, associate chief justice of Ontario) concluded that the MOE was aware of the issues at the Walkerton utility and had taken no action against it (O’Connor 2002a).

#### Politics Stream

The political context in Canada made it possible for policy changes to be effective. Firstly, the changes mainly all fell under a single Ministry, the MOE (Schwartz and McConnell 2009). Secondly, Canadian politics places high value on integrity, and therefore, a town failing to provide its residents with safe water was of high priority to the citizens and their representatives (Cote et al. 2017)—restoring public trust was imperative. Furthermore, Premier Mike Harris was not viewed in a very favourable light (even before the Walkerton incident) and many viewed his neoliberal policies of tax reductions and budget cuts to be a causal factor behind the Walkerton failure (Michaels et al. 2006; Schwartz and McConnell, 2009).

#### Policy Stream

The potential policy solutions to the Walkerton crisis stemmed from the causal factors which led to the incident: protecting water sources via a watershed-based approach, taking a risk-based approach to setting water quality standards, making said standards legally enforceable, stringent monitoring plans, mandatory training and certification of operators, enacting specific policies, such as the Safe Drinking Water Act, and much more (O’Connor 2002b).

#### Policy Window

The Walkerton incident posed a significant window of opportunity because the sudden illnesses and deaths (including visits to the emergency department and hospitalisations) due to the outbreak was a very clear focusing event, and the resulting policy solutions which were feasible and acceptable. The politics (including the national mood) were also conducive towards effective policy outcomes (Schwartz and McConnell 2009). As the judge said himself, the inquiry report was not recommending ‘radical reform’ but rather calling for best practices to be implemented (O’Connor 2002b, p. 2).

#### Policy Entrepreneurs

The main policy entrepreneur was Justice O’Connor, who headed the inquiry into the Walkerton case (Schwartz and McConnell 2009). Unlike the other policy entrepreneurs discussed here, O’Connor was—by virtue of his position in the system of governance in Ontario—automatically placed to be a policy entrepreneur. His investigation and subsequent report were well regarded and considered to have a high level of integrity (Schwartz and McConnell 2009). The report was praised for not hesitating to criticise the Harris government and its budget cuts, addressing the inaction of the MOE when it came to non-compliance, and objectively applying science and law in the inquiry process (Snider 2004).

There was also citizen action involved in this case. The Concerned Walkerton Citizens, a community group composed of over 500 members at the time who were among those who sought an inquiry, wanted to participate in the inquiry process to present their arguments, and ensure the local residents’ perspectives were represented (Canadian Environmental Law Association 2001). They were recognised as a party with full standing in all phases of the inquiry (O’Connor 2002a, b). This serves as an example of functioning democracy where not only is the government held accountable to its citizens, but the latter are allowed a meaningful role in the process. Thus, Walkerton is an example of a case where citizens undertook their social responsibility in the face of the crisis—and moreover, this citizen action was legitimised and recognised in the policy response process.

#### Policy Outputs and Outcomes

When the outbreak became apparent, the provincial government acted promptly (Schwartz and McConnell 2009), with municipal water systems being put under review, and an inquiry being set up in about 3 weeks (O’Connor 2002a). Regulatory procedures were immediately revamped in alignment with the findings of the inquiry. New legislations were passed, and guidelines were turned into enforceable standards (Schwartz and McConnell 2009). By 2002, the Ontario government enacted the Safe Drinking Water Act, and it introduced the Clean Water Act in 2005, both following from O’Connor’s recommendations (Michaels et al. 2006). The operators at the utility, brothers Frank and Stan Koebel, were prosecuted and pled guilty in 2004 (Keith 2010).

### Lead and *Legionella* in Flint, Michigan, USA, 2014

#### Problem Stream

In 2014, the city of Flint stopped purchasing water from the Detroit Water and Sewerage Department (DWSD), which abstracted and treated water from Lake Huron (Masten et al. 2016). Instead they changed their source to the Flint River, and treatment was done at the Flint Water Service Centre. Water quality of the Flint River was poor due to unregulated discharges by industries and municipalities (Masten et al. 2016). Residents found their water to be cloudy and discoloured, and there was a subsequent *Legionella* outbreak which ultimately led to 12 deaths (Masten et al. 2016). These issues were clear enough focusing events for the crisis, and were later further intensified by the discovery of elevated blood lead levels among Flint’s children (Hanna-Attisha et al. 2016).

Water treatment at the Flint plant was not effective since it had not been functional for almost 50 years,^2^ and moreover, it was understaffed (and the staff who were there were undertrained) (Masten et al. 2016). In addition, the corrosivity of the Flint River water was much higher than the Lake Huron water, resulting in the ageing lead-containing plumbing infrastructure of Flint to release lead into the drinking water (Masten et al. 2016).

#### Politics Stream

The politics around the Flint water crisis were counterintuitive to solving the crisis. Firstly, the state-appointed Emergency Manager (who was placed there to implement neoliberal austerity measures to cut costs in this impoverished post-industrial city (Fasenfest 2019)) ignored the citizens’ concerns regarding their drinking water (Davis et al. 2016; Denchak 2018). The Michigan Department of Environmental Quality (MDEQ) ignored reports on lead levels by the EPA and researchers from Virginia Tech, and continued to dismiss them even after the EPA report was leaked by the American Civil Liberties Union (Davis et al. 2016). The governor only approved the switch back to Detroit water after the results of a study on elevated blood lead levels in children were verified (which was also initially dismissed (Davis et al. 2016)), and drinking water in schools tested positive for high levels of lead as well (Denchak 2018).

#### Policy Stream

The main policy solution in the case of Flint was to switch back to the DWSD water supply (Davis et al. 2016). Other solutions include employing corrosion control measures, establishing a toxic exposure registry and improving screening of blood lead levels, reviewing the Emergency Manager Law, strengthening the Lead and Copper Rule to be less ambiguous, and replacing lead infrastructure (Davis et al. 2016).

#### Policy Window

The Flint water crisis did open a significant window of opportunity since there were several clear focusing events (undrinkable water, *Legionella* outbreak, high lead levels detected in water and in children’s blood) (Masten et al. 2016), and there were possible policy solutions. The difference here was that the policy solutions were not acceptable to the government authorities because they would incur high costs (approximately $12 million) (Davis et al. 2016).

#### Policy Entrepreneurs

The policy entrepreneurs in the Flint case were Marc Edwards and his research team from Virginia Tech who tested the lead levels in people’s homes, as well as the paediatrician Mona Hanna-Attisha and her team at the Hurley Medical Center who published a study on elevated blood lead levels among children that became a turning point in the government acknowledging the crisis (Masten et al. 2016). They made clear use of the policy window opened up by this crisis and presented robust empirical evidence that should have driven policy change and increased the sense of urgency. However, the political climate meant their evidence was not immediately taken into account.

Moreover, a further note must be made here to illustrate the complexity of this case. Despite the fact that it was the citizens of Flint (many of whom were African American) who first brought the issues to light (Johnson et al. 2018)—Lambrinidou (2018) points out that Edwards ended up becoming the heroic face of the response to the Flint crisis. Johnson et al. (2018) also show how the narrative around Flint has been largely controlled by those outside of the crisis, while African American residents were simply relegated to being victims of the crisis—downplaying their efforts towards finding a solution.

The citizens of Flint approached the authorities with their grievances in 2014 and 2015 in community and town hall meetings, and one of them even directly contacted the EPA (i.e. access) (Denchak 2018); citizens engaged with scientists, like Edwards in the collection of empirical data (i.e. resources) (Flint Water Study Updates 2015), and they had a network of grassroots activists and community organisations (i.e. strategy) (Johnson et al. 2018). And yet they were routinely dismissed by the relevant authorities (Davis et al. 2016). Thus, in Flint, despite being aware of the issues as well as concerted effort in the form of community organising and citizen science, residents were still side lined.

#### Policy Outputs and Outcomes

The switch back to Detroit Water did not occur until October 2015, by which time there had already been overwhelming evidence of lead in water and in children’s blood (Denchak 2018). State of emergency was then declared by the city, followed by the governor for the county, and then by the President (Davis et al. 2016). Moreover, in January 2015, the Michigan Department of Health and Human Services (MDHHS) asked the Genesee County Health Department to investigate the increased number of *Legionella* cases but dismissed the fact that it may be connected to the water source switch (Davis et al. 2016).^3^ It was not until a year later that the Governor of Michigan announced the *Legionella* outbreaks to the public (Denchak 2018). The Federal Emergency Management Agency then stepped in, and bottled water and water filters were distributed to households (Davis et al. 2016). Several lawsuits followed, including criminal charges against the state’s emergency managers who were responsible for the switch to Flint River as a cost-cutting measure, along with water treatment plant officials for tampering with evidence. The lead infrastructure is also gradually being replaced (Denchak 2018), although it has been delayed beyond its expected 2020 completion due to the COVID-19 pandemic (Hanna-Attisha et al. 2022). In 2019, the EPA proposed several revisions to improve the Lead and Copper Rule (US EPA 2019), which had previously left room for misinterpretation (Davis et al. 2016). Following that, in November 2021, a federal judge approved a settlement of $626 million for those affected by the Flint water crisis, much of which will be borne by Michigan state for disregarding the risks of switching to the Flint River (Guardian Staff and Agencies 2021).

### Chromium-6 in Hinkley, California, USA, 1987

#### Problem Stream

The Pacific Gas & Electric (PG&E) station in Hinkley, California used chromium-6 as an anti-corrosive on their compressors, and from 1952 to 1966 they dumped about 370 million gallons of chromium-containing wastewater into unlined ponds, which then percolated into the groundwater, contaminating private wells (Banks 2003; California Water Boards 2021a). The company did not notify the regional water authority of the contamination until 1987, after which they were required to clean up the chromium plume (Banks 2003). There was no clear focusing event in this case—since the contamination and exposure had occurred for years without Hinkley residents realising it.

This happened despite the fact that the same authority issued an order detailing waste discharge requirements for PG&E in 1972. The order stated that the discharge should be contained within land owned by PG&E; that the ‘discharges shall contain no trace elements, pollutants or contaminants, or combinations there­of in concentrations which are toxic or harmful to humans or to aquatic or terrestrial plant or animal life’; and listed maximum average concentrations and discharge rates for 14 substances (which the wastewater could not exceed) that included chromium-6 (California Regional Water Quality Control Board Lahontan Region 1972, p. 2).

#### Politics Stream

Interestingly, there was not much of a political interest in the Hinkley case initially: media attention was really only brought to this issue once Hollywood took an interest and made a movie about the events (Banks 2003). Before then, the general public did not have this small desert town or chromium-6 on their minds. Despite the media attention following the movie, there has still been a lack of policy response to chromium-6 in drinking water (with the federal government delaying adoption of new standards) due to manufactured uncertainty around the science of chromium-6 and its effect on health (Egilman 2006; Smith 2008; Sutton 2010).

For a detailed account of the attempts by corporate companies to deliberately manipulate the evidence regarding the carcinogenicity of chromium-6 (along with various other unethical research practices), see Egilman (2006). In brief, PG&E (through a consulting firm) hired a scientist, Dr Zhang—who had previously done a study in China which showed positive correlation between cancer and water containing chromium-6—to retract his previous conclusions in a new paper (Waldman 2005; Egilman 2006; Smith 2008). The paper was published in a well-reputed journal, but it was later revealed that Zhang did not agree to these altered conclusions, which his translator testified to in court (Egilman 2006). Although the journal retracted the paper (Smith 2008; Sutton 2010), the narrative of doubt had been set (Egilman 2006). This is in addition to the several scientists involved in chromium-6 research who have been involved with polluting industries and have demonstrated instances of not declaring conflicts of interest (Egilman 2006; Smith 2008).

#### Policy Stream

The major policy solutions in this case were the cleaning up of the chromium-6 plume in Hinkley (Steinpress and Ward 2001; Banks 2003) and setting a maximum contaminant level (MCL), i.e. a legally enforceable standard (Steinpress and Ward 2001; Sutton 2010).

#### Policy Window

In the case of the Hinkley water contamination, the policy window was not a significant one. The absence of a focusing event, lack of political interest, and more importantly the uncertainty created around the science of chromium-6 (Egilman 2006; Smith 2008), meant that implementation of any policy change has been slow, and not very effective.

#### Policy Entrepreneurs

Erin Brockovich (a law clerk at the firm representing the residents of Hinkley in their lawsuit against PG&E) played a crucial role in identifying a cluster of cancer cases in the town of Hinkley and building a case for them (Banks 2003). The lawsuit was successful, but this did not translate to major policy change due to the reasons discussed above.

The issue of the narrative being largely centred around those outside of the crisis also holds true for Hinkley and Erin Brockovich: although not a technical expert in the vein of Edwards, the legal clerk did become the face of the crisis in Hinkley, and the eponymous Hollywood movie went on to become a commercial and critical success. Although the community blamed PG&E for polluting their water, and believed this was the cause of their illnesses (Banks 2003)—and it was one particular resident who brought the law firm on in the first place because she was suspicious of PG&E’s actions (San Bernadino County Sun 2013)—the story primarily centres on Brokovich’s organisation of the community. Moreover, in the years since the settlement, the citizens of Hinkley have criticised the lawyers for lack of transparency in the settlement process, the fees they kept, and other questionable actions—the community does not see them as the heroes the media has made them out to be (Helmore 2000). Thus, in Hinkley (much like in Bangladesh and Flint) the concerned citizens were denied agency and side lined.

#### Policy Outputs and Outcomes

Three weeks after PG&E notified the Lahontan Regional Water Quality Board (LRWQB), they issued Cleanup and Abatement Order 6-87-160, which required PG&E to clean up the contaminated water (Banks 2003)—a task which is still ongoing (California Water Boards 2021a). As mentioned, when PG&E began the cleanup procedure, Hinkley residents became suspicious, and attributed many of their illnesses, including cancer, to the contamination—which led to around 650 residents filing a lawsuit (Pellerin and Booker 2000; Banks 2003). The lawsuit was settled outside of court and the residents were paid $333 million, which at the time was the largest ever settlement amount in the US (Banks 2003). However, beyond that, the policy response has been slow and the EPA has yet to adopt safe standard for chromium-6 despite its increasing recognition as a carcinogenic risk (Sutton 2010; He and Wu 2019)—including by the US Department of Health and Human Services (National Toxicology Program 2021). In fact, the current federal standard does not distinguish between chromium-6 and chromium-3, the latter of which is an essential nutrient—with the MCL for total chromium being 100 µg/L (US EPA 2021).

However, in 2014 California did adopt a separate MCL of 10 µg/L for chromium-6, but this was invalidated by a court order in 2017—thus the state currently has a total chromium MCL of 50 µg/L (California Office of Environmental Health Hazards Assessment 2022). The case was brought against the California Department of Public Health by the California Manufacturers and Technology Association, and according to the water board, the decision was not due to any conclusions on the adequacy of public health protection or the MCL value itself, but rather that ‘The court merely found that the department did not adequately document why the MCL was economically feasible’ (California Water Boards 2021b).

## Discussion

Policy responses can range between being proactive, reactive, and inactive (Damania et al. 2019). Furthermore, according to Boin et al. (2009), when a case of crisis exploitation occurs, depending on the nature of those involved, it could end in one of many ways. This includes a policy stalemate, politically imposed paradigm shift, politically imposed incremental adjustment, negotiated incremental adjustment, incremental substantive change, or major/swift rhetorical or symbolic change (Boin et al. 2009).

Within the context of this paper, effective policy change is defined as one where new policies are adopted (or older ones are updated) and then implemented—thus resulting in ending the immediate threat to water quality and working towards dealing with chronic outcomes (if any). Walkerton is an example of effective policy change. It involved an inquiry which was carried out in an efficient and timely manner, and immediate implementation of many of the recommendations, such as updating the regulatory procedures and turning what were formerly guidelines into enforceable standards. According to Boin et al. (2009), Walkerton is a case of a ‘major policy adjustment’ (p. 93).

Within the context of this paper, ineffective policy change would be when a new or updated policy is put forth much too late, not implemented, or was not adequate or appropriate to deal with the crisis. Examples of ineffective policy change would be Bangladesh’s National Policy for Arsenic Mitigation (2004) being published over a decade after the arsenic crisis was officially recognised; the distribution of bottled water and filters in Flint several months after citizens began complaining about their water quality; and the US EPA delaying the setting of chromium-6 standards due to the manufactured uncertainty around it. If Boin et al.’s (2009) crisis exploitation framework were applied here, Bangladesh and Flint would likely be examples of negotiated incremental change, whereas Hinkley is a case of policy stalemate.

From these cases, it is evident that the acute/chronic nature of the contamination, along with factors, such as the social and political context, visibility of outcomes and affected populations, public/media attention, as well as accountability and placement of blame, plays important roles in influencing the policy process. These are discussed in more detail below.

### Acute Versus Chronic Nature of Events

One key point of comparison is the temporal nature of these events. The terms acute and chronic can be used to describe both the exposure and the outcome (Table 3): exposure here refers to the presence of the contaminant in drinking water at potentially harmful concentrations; outcome refers to the negative health impacts originating from the exposure.

**Table 3:** Combinations of acute and chronic exposures and outcomes

	Acute outcome	Chronic outcome
Acute exposure	Flint, Walkerton	Flint, Walkerton
Chronic exposure		Bangladesh, Flint, Hinkley

In Walkerton the exposure and outcome were both acute; however, the literature does indicate that chronic health issues arose (Clark et al. 2010). In comparison, in Flint, exposure and outcomes (illness and death) related to *Legionella* were acute, but the exposure and outcome (developmental impairments) related to lead were chronic. Hinkley and Bangladesh are both cases where the exposure was chronic (and unknown to those experiencing it for a number of years), and the resulting health outcomes are also chronic—mainly resulting in cancers and other long-term diseases.

The period of subclinical disease seems to play an important role in the policy outcomes. This is known as the ‘incubation period’ for infectious diseases and ‘latency period’ for chronic diseases (CDC 2012). Water crisis events which involve contaminants with a longer subclinical period also involved longer policy response times (Table 4). Thus, it is not only the chronic nature of disease but also the time taken for health outcomes to become apparent that has an important effect on how the crisis is dealt with.

**Table 4:** Incubation or latency period for contaminants present in events

Exposure	Incubation/latency period	Policy response time
*E. coli O157:H7*	**3–8 days** (WHO 2018)	3 weeks to 2 years (Walkerton)
*Campylobacter*	**2–5 days** (CDC 2019)	
*Legionella*	**2–14 days** (longer periods in rare cases) (CDC 2021a)	6 months to 1½ years (Flint)
Lead	Can be **indefinitely subclinical** depending on dose^a^	1½ to 5 years (Flint)
Chromium-6	Unclear for exposure through drinking water; around **10–18 years** based on available evidence from China (Beaumont et al. 2008; Smith and Steinmaus 2009)^b^	3 weeks to 27 years (Hinkley)
Arsenic	**6–9 years** (depending on dose and other host factors) for skin lesions (WHO 2005)	13–20 years (Bangladesh)

^a^This because lead is not considered safe at any dose for the developing brain (WHO 2010). The previous guideline for tolerable intake was 25 µg/kg of body weight per week for infants and children, but this was withdrawn in 2010 since it was inadequate to protect against loss of IQ (WHO 2010)

^b^The main limitation is that the observation period was limited to eight years, covering 10–18 years of exposure (Smith and Steinmaus 2009); environmental carcinogens can have a latent period not only of more than 15 years but can also be shorter depending on the toxic agent and the organ (Beaumont et al. 2008)

For the purpose of this paper, the time taken for policy response has been counted as the instances where a substantial mitigation effort was put forth—thus not counting initial boil water advisories (where applicable). The Walkerton inquiry was established in 3 weeks, with the reports for the first and second parts being published 8 months and 2 years after the incident, respectively (O’Connor 2002a, b). The MOE began revising its guidelines according to the preliminary findings of the inquiry (Schwartz and McConnell 2009), and the Ontario government ensured drinking water safety in the interim period via Operation Clean Water and the temporary Drinking Water Protection Regulation which set legally binding standards (Canadian Environmental Law Association 2011). So even though these changes became fully enshrined in law when the Safe Drinking Water Act was enacted later in 2002 and the Clean Water Act in 2005, substantial policy change had already taken place in the 2 years it took to conduct the inquiry and publish its full findings and recommendations.

In Flint, the empirical evidence for lead was accepted by the MDEQ, the Governor of Michigan approved $9 million to tackle the water crisis, and the water supply was switched back to DWSD a year and a half after the issues had become apparent (Denchak 2018). The Flint case provides a contrast because the contaminants included both *Legionella* which has a short subclinical phase (2–10 days) as well as lead, which can be indefinitely subclinical at lower doses (WHO 2010). The policy response in Flint with regard to lead and *Legionella* was somewhat separate. Both issues became apparent by June 2014 (Denchak 2018). The MDHHS ordered an investigation into the *Legionella* outbreak in January 2015 (Davis et al. 2016), but it was not until a year later that the *Legionella* outbreaks were announced to the public (Denchak 2018). Thus, the response timeline for *Legionella* is considered 6 months to 1½ years here. The first lead advisory was issued in September 2015, along with free tests being offered to residents of Flint (Denchak 2018). By 2019 the city made a commitment to replace the remaining lead pipes (Denchak 2018), and revisions to strengthen the Lead and Copper Rule were proposed (US EPA 2019). Thus, the lead response timeline is considered approximately 1½–5 years.

The timeline for Hinkley is also complex. The start of the policy response was December 1987, 3 weeks after PG&E notified the regional water authority of the contamination. As mentioned before, California adopted the (now invalidated) chromium-6 standard in 2014. Thus the policy response time for Hinkley was 3 weeks to 27 years. For Bangladesh, the start of the crisis is being counted as the identification of the first arsenicosis patients in 1984 (Atkins et al. 2007). The time taken for policy response is being counted as the 1997 World Bank funding for BAMWSP as well as DPHE and UNICEF’s well screening, up to 2004 when the National Policy for Arsenic Mitigation was published (i.e. 13–20 years) (Atkins et al. 2007). However, it is important to note that the ‘end point’ is not meant to be a definitive indication of the end of the crisis but rather the point where major policy decisions tapered off.

In relation to the subclinical period, a note should be made on access to healthcare services in the four different contexts. As shown in Table 2, Ontario, Michigan and California all outperform Bangladesh on this indicator (i.e. physicians per 1000 population). During the initial recognition of the arsenic crisis, this was 0.2 per 1000 people nationally, and has since only increased to 0.6 as of 2019 (The World Bank 2022). Moreover, according to a study by Ahmed et al. (2013) this proportion was 16.5 times lower in rural areas than urban ones. In a study by Ahmad et al. (2007), 43.2% of respondents (*n* = 750) did not know about healthcare facilities available to them for treatment of arsenicosis symptoms. For those who had sought treatment, common issues cited included irregular medicine supply, transportation issues, and lack of available doctors, among others (Ahmad et al. 2007).

### Social and Political Context

It is important to consider the type of government that the citizens in each of these contamination events are accustomed to. Canada is considered a full democracy (ranking 5th in the world), and therefore, its citizens are used to a certain level of civil liberties, faith in functioning of the government, and a culture of political participation (The Economist Intelligence Unit 2021). There are expectations by citizens for the government they elected to provide services to a certain standard, especially basic needs, such as water. The Walkerton events resulted in a loss of trust and faith in the authorities (Schwartz and McConnell 2009), and for Canadians this was considered a threat to their sense of identity (Cote et al. 2017).

On the other hand, the US is considered a flawed democracy (Table 2). It has a long and complex history with environmental injustice (Brulle and Pellow 2006), wherein poorer and minority ethnic communities have been disproportionately exposed to hazardous materials. Moreover, there are functions within the system of governance in the US which circumvent accountability to the electorate—and the emergency managers are a prime example of that (Fasenfest 2019). Flint is widely considered a case of environmental racism (Campbell et al. 2016), not just due to the disparate impacts of lead exposure on children of colour (Hanna-Attisha et al. 2016, 2022) but also because it is a ‘result of systemic racism that was built into the foundation and growth of Flint’ (Michigan Civil Rights Commission 2017, p. 2). It is reflected in the history of Flint’s industry, employment, segregated housing, and education (Michigan Civil Rights Commission 2017), as well as in the erasure of the voices of concern in the African American community (Johnson et al. 2018).

For Bangladesh, being a hybrid regime means there are authoritarian aspects to the system of governance, and in addition, much of the affected populations are the rural poor whose voices are less likely to be taken into account in such a centralised government. As Atkins et al. (2007) pointed out there was ‘surprisingly little debate or publicly-expressed anger’ regarding the arsenic issue (p. 164).

### The Politics of Visibility

Visibility plays an important role in each of these water contamination events. This involves both the visibility of the outcomes and of the population affected. In the case of Walkerton, the effects were immediate. In terms of the exposed population, Walkerton is a small rural town in Canada which was otherwise no different from its many other small rural towns (Cote et al. 2017). However, the media attention was persistent and the fear and anxiety resounded across the country (Cote et al. 2017). The reaction to Walkerton, however, is a stark contrast to the many First Nations communities in Canada that have a chronic lack of access to safe drinking and domestic water (Galway 2016). There are still 36 long-term drinking water advisories across 29 communities in place as of January 2022 (with 127 having been lifted since November 2015) (Government of Canada 2022).

The health effects of lead, chromium, and arsenic, however, are much less visible and may take a while to manifest. The cases in Flint, Hinkley, and Bangladesh also have the commonality of the affected populations not counting among the ‘social elite’, in part due to their economic status—with Flint being an impoverished post-industrial city, Hinkley an unincorporated town in the desert, and Bangladesh mainly having poorer rural areas affected by the arsenic crisis.

As shown in Table 2, Flint’s GDP is much lower than the national level, and Hinkley is largely considered a ‘ghost town’ (Pearl 2015; Genecov 2019). In Bangladesh, the percentage of GDP contribution from sectors mainly associated with rural areas (i.e. agriculture, forestry and fishing) has been on a steep decline since 1993—decreasing from 27.3% then to 12.9% in 2020 (The World Bank 2022). About 35 million people were estimated to be exposed to arsenic above the Bangladesh standard of 50 µg/L and 57 million above the WHO guideline of 10 µg/L (Kinniburgh and Smedley 2001)—approximately 28–46% of the 2001 population (see Table 2). Despite these large numbers, the arsenic-affected population of Bangladesh are still among the rural poor whose contribution to the national GDP has been dwindling—thus decreasing their visibility.

### Public and Media Attention

The public and media attention was immediate and intense in the case of Walkerton (Cote et al. 2017), and eventual in the case of Flint (Jackson 2017). As mentioned before, the general population of the US was not aware of Hinkley and its chromium-6 contamination up until the movie was released—and it soon lost steam afterwards. The Bangladesh arsenic crisis has received more diffused attention, with the crisis gaining media attention initially but then declining since the mid-2000s (Fischer 2019). Attention to the issue surges every now and then, with one recent example being the publication of the 2016 Human Rights Watch report on arsenic in Bangladesh. This is of course more expected with chronic events, such as Hinkley and Bangladesh—however, the content of the outcries has been quite different from that of Walkerton and even Flint—in the case of Bangladesh, the relevant authorities’ failure to meet a need as basic as water was not probed, and in Hinkley the attention was focused on the uncertainties regarding the contaminants.

### Accountability and Blame

In the case of Walkerton, blame was accepted and those who were responsible were held accountable. This never occurred in Bangladesh—UN organisations are immune from legal process (United Nations 1946), and the government has not been sued for the arsenic crisis. The only lawsuit which did take place did not turn out to be fruitful or productive.

Hinkley, on the other hand, is one of the most famous cases of a lawsuit being settled for such a large sum of money. However, that did not come to much, as half of it was kept for legal fees, and the other half was distributed among plaintiffs who comprised about a third of the population of Hinkley at the time (Genecov 2019). More importantly, PG&E has still not finished cleaning up the chromium plume (California Water Boards 2021a) and in fact has bought up much of the housing along the edge of the plume and bulldozed them, which has resulted in Hinkley’s population decreasing significantly, and those who still remain are still struggling with the quality of their water (Genecov 2019).

In Flint, fifteen state and local officials had criminal charges filed against them (Smith 2019). Among them, seven took plea deals, and the other eight (a group mainly comprised of the highest-ranking officials involved in the crisis) had all charges dropped in 2019 due to concerns about all evidence not being pursued (Smith 2019). The investigation was restarted with the possibility of refiling the charges (Smith 2019), and early in 2021 nine officials were charged with various offences (Gray and Bosman 2021). This included wilful neglect of duty for the former Michigan governor and involuntary manslaughter for two former health officials (Gray and Bosman 2021). And as mentioned previously, the state of Michigan is responsible for most of the $626 million settlement for Flint residents (Guardian Staff and Agencies 2021).

### How Do These Factors Interact?

Thus far, it seems as though a ‘positive outcome’ is needed with regard to most of the factors discussed above for the resulting policy changes to be effective and the water quality issue to be resolved (Table 5). It is understandable for cases, like Bangladesh and Hinkley, where there were more hindrances than favourable conditions, why the outcome is then negative. Flint, however, could have been expected to reach a resolution sooner—but the denial from government officials and institutions despite the overwhelming evidence was key to slowing it down. Moreover, according to Hanna-Attisha et al. (2022), ‘The recommended practice of primary prevention, the proactive detection of lead in and removal from the environment before children are exposed, has yet to be realized’ (p. 72).

**Table 5:**
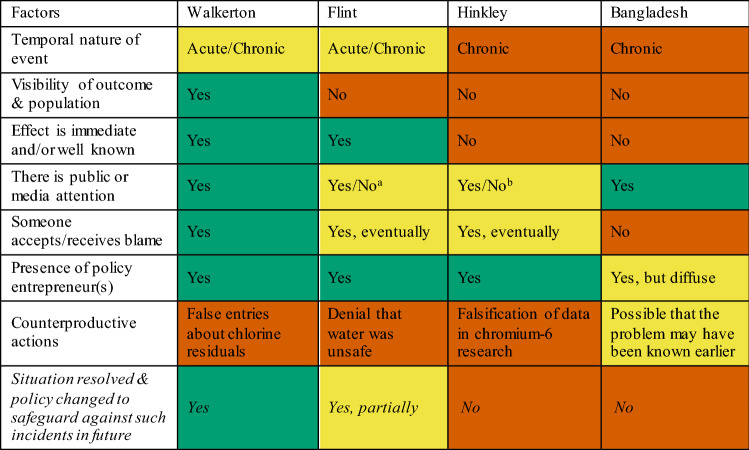
Variables that influenced the effectiveness of the policy change (orange indicates a barrier to change, green indicates incentives to change, yellow indicates an unclear relationship)

^a^Initially was going unnoticed, but the media (Jackson 2017) and other political movements such as Black Lives Matter (Rakia 2016) eventually took notice

^b^Media attention mainly came about in the aftermath of the movie (Banks 2003)

It is also important to note that cases that seem perfectly successful are not exactly so—it just so happened that most things worked out favourably. For example, in Walkerton the utilities operator made false data entries about chlorine residuals, and concealed facts from the local public health authority (which otherwise could have prevented 300–400 cases of illness) (O’Connor 2002c).

### Limitations and Further Opportunities for Policy Analyses of Crisis Events

The Multiple Streams Framework is a useful tool with which comparative studies on other water (as well as wider environmental) crises can be conducted. There is a need for more research on these complex policy processes in order to further support the use of MSF for comparative work. For example, further research on complex case studies can be conducted: such as acute epidemic cases, like the 2010 Haiti cholera outbreak which was further complicated by the 7.0 magnitude earthquake preceding it, and the role of the UN in the governance of the country at the time (Komlan 2019); or even longer-term contamination cases than arsenic, such as South Africa’s more than century-long history with acid mine drainage from gold mining in the Witwatersrand Basin (Harvard Law School International Rights Clinic 2016). These would help better understand different policy impacts of water quality crises.

Water contamination crises are common globally; however, there is limited published evidence on crises that both result from human action and cause harm to people. This lack of available literature is one of the key limitations of this research, restricting the case studies to older cases and cases primarily in developed contexts. There was insufficient data on recent water contamination crises and crises in developing countries to expand the case studies. The wider evidence on water contamination crises did not fully addresses our search criteria. For example, fluoride is widespread in groundwater in Ethiopia (Demelash et al. 2019); however, it is solely geogenic and not due to human action. Also, there are examples of crises where, while they posed a threat to public health, there was no evidence that they affected human health (unlike the case studies discussed in this paper), e.g. the 1998 *Cryptosporidium* and *Giardia* contamination in Sydney (McConnell 2008) or the 2007 cyanobacterial bloom in Lake Taihu in China (Qin et al. 2010). In other cases, there was inadequate literature on the actual policy response (or lack thereof) to the contamination crisis, e.g. cyanide spillages in Ghana (Amegbey and Adimado 2003). Although limited to four events, this paper provides a novel comparative analysis of water quality crises revealing valuable insights into the policy process.

## Conclusion

Acute water contamination crises, that have a visible outcome on a vocal population, supported by media attention, strong science, and policy entrepreneur, can create policy change. However, these conditions are not present for millions around the world who experience unsafe water. And as the cases highlight, water contamination crises are experienced even in areas where safe water has become the accepted norm. This begs the question: do we need to rethink or restructure the way water quality issues are dealt with? Methodologies exist to compare the health burden from acute and chronic health outcomes, such as Disability Adjusted Life Years to compare diarrheal deaths with chronic arsenicosis, but do not motivate political will or support accountability. It is clear from the above analysis that many things have to go right in order for the policy response to be effective (as in the case of Walkerton), and any counterproductive actions can prove to be quite dangerous (as in the case of Flint and Hinkley). In cases, such as Hinkley and Bangladesh, there were too many factors in play that worked against a timely and effective solution.

In all cases, the Multiple Streams Framework methodology has helped demonstrate there was a clear recognition that a crisis situation was occurring—reflected either through the public or media attention, the presence of policy entrepreneurs, or someone eventually taking the blame. In a case like Bangladesh—where the affected population is not easily visible, when there is nobody to be held accountable, and with a diffuse policy entrepreneurship taking the reins—what can be done to ensure the threat to public health is removed and water quality is regulated in a way that prevents any future occurrences of such contamination events?

Damania et al. (2019) describe water quality as a ‘wicked problem’—i.e. one which is complex and difficult to solve because there is no obvious single solution—however, this is not the whole story. Great advances have been made in water quality and drinking water safety in many developed economies. As Walkerton illustrates, water quality issues are not *impossible* to solve, but a range of complex conditions have to be met in order to reach a successful solution.

When a water quality crisis occurs, the larger context of politics, financing, and public health need to be taken into account to understand the trajectory of the crisis. Flint was going through a major economic crisis and its water supply system ended up taking the hit. In the case of Bangladesh, surface water pathogens were largely responsible for the major waterborne disease burden, the response to which led to the arsenic crisis. Hinkley provides an important example of competing interests. In Walkerton, *E. coli* O157:H7 was the face of the crisis but it was the lack of regulation which was recognised as the actual issue. Thus, safeguarding water quality for the future requires systemic changes that factors in potential risks, including ones which have not yet been faced.

The scientific evidence linking water quality and health outcomes, and acceptance thereof, remains important. For diarrhoeal diseases, transmission via water is well understood and remains the primary focus of water safety management, with *E. coli* the primary indicator of drinking water safety (Charles et al. 2020). In many areas, our understanding of the links between water quality and health continues to grow and highlight new potential health crises: from the chronic impacts of infectious diseases on child development through stunting (Budge et al. 2019) to new evidence on the harms from known contaminants, such as manganese (WHO 2021), also of widespread concern in Bangladesh (Kinniburgh and Smedley 2001) with growing evidence of neurotoxicity in children (Khan et al. 2011). Furthermore, arsenic itself is a global threat, with a recent risk map estimating a potential 94–220 million people exposed to arsenic above WHO guideline levels (Podgorski and Berg 2020). In addition, there are many emerging water quality issues—such as microplastics, perfluorinated compounds, and antimicrobial resistance (Stefanakis and Becker 2016; Damania et al. 2019). Water quality crises will continue to grow, with new approaches needed to help leverage them for change.

## Data Availability

Data sharing not applicable to this article as no datasets were generated or analysed during the current study.

## Footnotes

1 Not to be confused with the earlier survey which they were sued for, the result of the later project (Kinniburgh and Smedley 2001) is to date one of the most extensive databases of arsenic hydrological data in Bangladesh.

2 E.g. monitoring plans were not adequate; filter head loss metres were not made operational; equipment for measuring chlorine residuals was not installed; there were neither corrosion control plans nor corrosion control equipment installed (Masten et al. 2016).

3 According to a study by Zahran et al. (2018), the incidence of Legionnaire’s Disease increased after the switch to Flint River water and decreased after the switch back in October 2015.

## References

[CR1] Ahmad SA, Sayed MHS, Khan MH (2007). Sociocultural aspects of arsenicosis in Bangladesh: community perspective. J Environ Sci Health A.

[CR2] Ahmed SM, Evans TG, Standing H, Mahmud S (2013). Harnessing pluralism for better health in Bangladesh. Lancet.

[CR3] Amegbey NA, Adimado AA (2003). Incidents of cyanide spillage in Ghana. Miner Process Extr Metall.

[CR5] Atkins PJ, Hassan MM, Dunn CE (2006). Toxic torts: arsenic poisoning in Bangladesh and the legal geographies of responsibility. Trans Inst Br Geogr.

[CR4] Atkins P, Hassan M, Dunn C (2007). Poisons, pragmatic governance and deliberative democracy: the arsenic crisis in Bangladesh. Geoforum.

[CR6] Bangladesh Bureau of Statistics (2011) Population & housing census 2011: national volume 2 union statistics. http://203.112.218.65:8008/WebTestApplication/userfiles/Image/National%20Reports/Union%20Statistics.pdf. Accessed 29 May 2022

[CR7] Banks S (2003). The “Erin Brockovich Effect”: how media shapes toxics policy. Environs.

[CR8] Beaumont JJ, Sedman RM, Reynolds SD (2008). Cancer mortality in a Chinese population exposed to hexavalent chromium in drinking water. Epidemiology.

[CR9] Birkland TA (1998). Focusing events, mobilization, and agenda setting. J Public Policy.

[CR10] Boin A, ’T Hart P, McConnell A (2009). Crisis exploitation: political and policy impacts of framing contests. J Eur Public Policy.

[CR11] Brulle RJ, Pellow DN (2006). Environmental justice: human health and environmental inequalities. Annu Rev Public Health.

[CR12] Budge S, Parker AH, Hutchings PT, Garbutt C (2019). Environmental enteric dysfunction and child stunting. Nutr Rev.

[CR13] Cairney P, Jones MD (2016). Kingdon’s multiple streams approach: what is the empirical impact of this universal theory?. Policy Stud J.

[CR14] Caldwell BK, Caldwell JC, Mitra SN, Smith W (2003). Searching for an optimum solution to the Bangladesh arsenic crisis. Soc Sci Med.

[CR15] California Department of Health Services (2000) Public Health Assessment for Pacific Gas And Electric (a/k/a Hinkley Site). https://www.waterboards.ca.gov/lahontan/water_issues/projects/pge/docs/hinkley/pha_122000.pdf. Accessed 31 Jan 2022

[CR16] California Office of Environmental Health Hazards Assessment (2022) Chromium-hexavalent. https://oehha.ca.gov/chemicals/chromium-hexavalent. Accessed 31 Jan 2022

[CR17] California Regional Water Quality Control Board Lahontan Region (1972) Board Order No. 6-72-44 waste discharge requirements for pacific gas and electric company Hinkley Compressor Station San Bernardino County. https://www.waterboards.ca.gov/lahontan/water_issues/projects/pge/cao/docs/refs/1_6_72_44.pdf. Accessed 31 Jan 2022

[CR18] California Water Boards (2021a) PG&E Hinkley chromium cleanup. https://www.waterboards.ca.gov/lahontan/water_issues/projects/pge/. Accessed 31 Jan 2022

[CR19] California Water Boards (2021b) Chromium-6 drinking water MCL. https://www.waterboards.ca.gov/drinking_water/certlic/drinkingwater/Chromium6.html. Accessed 31 Jan 2022

[CR20] Campbell C, Greenberg R, Mankikar D, Ross R (2016). A case study of environmental injustice: the failure in Flint. Int J Environ Res Public Health.

[CR21] Canadian Environmental Law Association (2001) Walkerton inquiry, part 1A and 1B: final argument on behalf of the Concerned Walkerton Citizens. https://cela.ca/walkerton-inquiry-part-1a-and-1b-final-argument-on-behalf-of-the-concerned-walkerton-citizens/. Accessed 31 Jan 2022

[CR22] Canadian Environmental Law Association (2011) FAQs: safe drinking water act, 2002, and its regulations. https://cela.ca/faqs-safe-drinking-water-act-2002-and-its-regulations/. Accessed 31 Jan 2022

[CR23] Canadian Institute for Health Information (2022) Scott’s Medical Database (SMDB). In: Physicians in Canada. https://www.cihi.ca/en/physicians-in-canada. Accessed 29 May 2022

[CR24] CDC (2012) Introduction to epidemiology: natural history and spectrum of disease. In: Principles of epidemiology in public health practice, third edition: an introduction, 3rd edn. CDC, Atlanta

[CR25] CDC (2019) Campylobacter (Campylobacteriosis): information for health professionals. https://www.cdc.gov/campylobacter/technical.html. Accessed 31 Jan 2022

[CR26] CDC (2021a) Legionella (Legionnaires’ Disease and Pontiac Fever): clinical features. https://www.cdc.gov/legionella/clinicians/clinical-features.html. Accessed 31 Jan 2022

[CR27] CDC (2021b) Flint lead exposure registry. https://www.cdc.gov/nceh/lead/programs/flint-registry.htm. Accessed 31 Jan 2022

[CR29] Charles KJ, Nowicki S, Thomson P, Bradley D (2019). Water and health: a dynamic, enduring challenge. Water science, policy, and management: a global challenge.

[CR28] Charles KJ, Nowicki S, Bartram JK (2020). A framework for monitoring the safety of water services: from measurements to security. npj Clean Water.

[CR30] Clark WF, Sontrop JM, Macnab JJ (2010). Long term risk for hypertension, renal impairment, and cardiovascular disease after gastroenteritis from drinking water contaminated with *Escherichia coli* O157:H7: a prospective cohort study. BMJ.

[CR31] Cohen DT (2007) Population distribution inside and outside incorporated places: 2000. https://www.census.gov/library/working-papers/2007/demo/POP-twps0082.html. Accessed 31 Jan 2022

[CR32] Cote SA, Ross HC, David K, Wolfe SE (2017). Walkerton revisited: how our psychological defenses may influence responses to water crises. Ecol Soc.

[CR33] Damania R, Rodella A-S, Russ J, Zaveri E (2019). Quality unknown: the invisible water crisis.

[CR34] Davis MM, Kolb C, Reynolds L et al (2016) Flint Water Advisory Taskforce: Final Report. https://www.michigan.gov/-/media/Project/Websites/formergovernors/Folder6/FWATF_FINAL_REPORT_21March2016.pdf?rev=284b9e42c7c840019109eb73aaeedb68. Accessed 25 Sep 2022

[CR35] Demelash H, Beyene A, Abebe Z, Melese A (2019). Fluoride concentration in ground water and prevalence of dental fluorosis in Ethiopian Rift Valley: systematic review and meta-analysis. BMC Public Health.

[CR36] Denchak M (2018) Flint water crisis: everything you need to know. In: Natural Resources Defense Council. https://www.nrdc.org/stories/flint-water-crisis-everything-you-need-know. Accessed 31 Jan 2022

[CR37] DPHE and JICA (2010) Situation analysis of arsenic mitigation 2009. Local Government Division, Government of Bangladesh; Department of Public Health Engineering (DPHE); JICA Bangladesh, Dhaka

[CR38] Egilman D (2006). Corporate corruption of science—the case of chromium(VI). Int J Occup Environ Health.

[CR39] Fasenfest D (2019). A neoliberal response to an urban crisis: emergency management in Flint, MI. Crit Sociol.

[CR40] Fischer A (2019). Constraining risk narratives: a multidecadal media analysis of drinking water insecurity in Bangladesh. Ann Am Assoc Geogr.

[CR41] Flint Water Study Updates (2015) Lead testing results for water sampled by residents. http://flintwaterstudy.org/information-for-flint-residents/results-for-citizen-testing-for-lead-300-kits/. Accessed 31 Jan 2022

[CR42] Fox WF, Menon B (2008) Decentralization in Bangladesh: change has been illusive. In: International Studies Program. Working Paper. https://icepp.gsu.edu/files/2015/03/ispwp0829.pdf. Accessed 31 Jan 2022

[CR43] Galway L (2016). Boiling over: a descriptive analysis of drinking water advisories in first nations communities in Ontario, Canada. Int J Environ Res Public Health.

[CR44] Genecov M (2019) Still toxic after all these years. In: Grist. https://grist.org/Array/the-true-story-of-the-town-behind-erin-brockovich/. Accessed 31 Jan 2022

[CR45] Government of Canada (2022) Ending long-term drinking water advisories. In: Indigenous Service Canada. https://www.sac-isc.gc.ca/eng/1506514143353/1533317130660. Accessed 31 Jan 2022

[CR46] Gray K, Bosman J (2021). Nine Michigan Leaders Face Charges in Water Crisis That Roiled Flint.

[CR47] Guardian Staff and Agencies (2021) ‘We’ve made history’: Flint water crisis victims to receive $626m settlement. The Guardian, London. https://www.theguardian.com/us-news/2021/nov/10/weve-made-history-flint-water-crisis-victims-to-receive-626m-settlement?fbclid=IwAR2_sPyfYy5w3Hjf29C7-VQfRSqTwYMcD17Cv-VcZqKWMEHbPCygiSWbqG0. Accessed 25 Sep 2022

[CR49] Hanchett S, Nahar Q, Van Agthoven A (2002). Increasing awareness of arsenic in Bangladesh: lessons from a public education programme. Health Policy Plan.

[CR48] Hanchett S, Monju T, Akhter K (2014). Water culture in South Asia: Bangladesh perspectives.

[CR50] Hanna-Attisha M, Lachance J, Sadler RC, Schnepp AC (2016). Elevated blood lead levels in children associated with the flint drinking water crisis: a spatial analysis of risk and public health response. Public Health.

[CR51] Hanna-Attisha M, O’Connell L, Saxe-Custack A (2022). Turning crisis into opportunity. J Pediatr Health Care.

[CR52] Harvard Law School International Rights Clinic (2016). The cost of gold: evironmental, health, and human rights consequences of gold mining in South Africa’s West and Central Rand.

[CR53] He S, Wu J (2019). Hydrogeochemical characteristics, groundwater quality, and health risks from hexavalent chromium and nitrate in groundwater of Huanhe Formation in Wuqi County, Northwest China. Expo Health.

[CR54] Helmore E (2000) Poisoned town condemns its movie-heroine lawyer. In: Guard. https://www.theguardian.com/world/2000/apr/16/film.artsreviews. Accessed 30 May 2022

[CR55] Human Rights Watch (2016) Nepotism and neglect: the failing response to arsenic in the drinking water of Bangladesh’s rural poor. https://www.hrw.org/sites/default/files/report_pdf/bangladesh0416web_1.pdf. Accessed 25 Sep 2022

[CR56] Jackson DZ (2017). Environmental justice? Unjust coverage of the Flint water crisis.

[CR57] Johnson JE, Key K, Bailey S (2018). Credit where credit is due: race and recognition in responses to the drinking water crisis in flint. Prog Community Health Partn Res Educ Action.

[CR58] Johnston RB, Sarker MH (2007). Arsenic mitigation in Bangladesh: national screening data and case studies in three upazilas. J Environ Sci Health A.

[CR59] Jones MD, Peterson HL, Pierce JJ (2016). A river runs through it: a multiple streams meta-review. Policy Stud J.

[CR60] Karim MF, Mimura N (2008). Impacts of climate change and sea-level rise on cyclonic storm surge floods in Bangladesh. Glob Environ Change.

[CR61] Keith N (2010). Sentencing the corporate offender: from deterrence to corporate social responsibility. Crim L Q.

[CR62] Khan K, Factor-Litvak P, Wasserman GA (2011). Manganese exposure from drinking water and children’s classroom behavior in Bangladesh. Environ Health Perspect.

[CR63] Kinniburgh DG, Smedley PL (2001) Arsenic contamination of groundwater in Bangladesh vol 2: final report

[CR64] Komlan A (2019). The Haiti cholera outbreak and peacekeeping paradoxes. Peace Rev.

[CR65] Lambrinidou Y (2018). When technical experts set out to “Do Good”: deficit-based constructions of “the Public” and the moral imperative for new visions of engagement. Mich J Sustain.

[CR66] Li P, Wu J (2019). Drinking water quality and public health. Expo Health.

[CR67] Masten SJ, Davies SH, McElmurry SP (2016). Flint water crisis: what happened and why?. J Am Water Works Assoc.

[CR68] McConnell A (2008). Ripples not waves: a policy configuration approach to reform in the wake of the 1998 Sydney water crisis. Governance.

[CR69] Michaels S, Goucher NP, McCarthy D (2006). Policy windows, policy change, and organizational learning: watersheds in the evolution of watershed management. Environ Manag.

[CR70] Michigan Civil Rights Commission (2017) The flint water crisis: systemic racism through the lens of Flint. https://www.michigan.gov/documents/mdcr/VFlintCrisisRep-F-Edited3-13-17_554317_7.pdf. Accessed 31 Jan 2022

[CR71] National Center for Health Statistics (1990) Health, United States, 1989 and prevention profile. In: Hyattsville, MD Public Health Services https://www.cdc.gov/nchs/data/hus/hus89acc.pdf. Accessed 29 May 2022

[CR72] National Center for Health Statistics (2018) Health, United States, 2017: with special feature on mortality. National Center for Health Statistics, Hyattsville. https://www.cdc.gov/nchs/data/hus/hus17.pdf. Accessed 29 May 2022 30702833

[CR73] National Toxicology Program (2021) Chromium hexavalent compounds. In: Report on carcinogens, 15th edn. U.S. Department of Health and Human Services, Public Health Service, Research Triangle Park

[CR74] Nowicki S, Koehler J, Charles KJ (2020). Including water quality monitoring in rural water services: why safe water requires challenging the quantity versus quality dichotomy. npj Clean Water.

[CR75] O’Connor DR (2002a) The Walkerton inquiry—part one: report of the Walkerton inquiry, The Events of May 2000 and Related Issues

[CR76] O’Connor DR (2002b) The Walkerton inquiry—part two: report of the Walkerton inquiry, The Events of May 2000 and Related Issues

[CR77] O’Connor DR (2002c) Part one: a summary report of the Walkerton inquiry: the Events of May 2000 and Related Issues summary of the report

[CR78] Open Data Network (2022) GDP per capita Data for Flint Metro Area (MI)—Gross Domestic Product on the Open Data Network. https://www.opendatanetwork.com/entity/310M200US22420/Flint_Metro_Area_MI/economy.gdp.per_capita_gdp?year=2014. Accessed 29 May 2022

[CR79] Panday PK (2011). Local government system in Bangladesh: how far is it decentralised?. Lex Localis J Local Self-Gov.

[CR80] Pearce F (2001). Bangladesh’s arsenic poisoning: who is to blame?. UNESCO Cour.

[CR81] Pearl M (2015) The Town Erin Brockovich rescued is basically a ghost town now. VICE Mag. https://www.vice.com/en/article/xd7qvn/the-town-erin-brockovich-rescued-is-now-almost-a-ghost-town-992. Accessed 25 Sep 2022

[CR82] Pellerin C, Booker SM (2000). Reflections on hexavalent chromium: health hazards of an industrial heavyweight. Environ Health Perspect.

[CR83] Podgorski J, Berg M (2020). Global threat of arsenic in groundwater. Science.

[CR84] Prudham S (2004). Poisoning the well: neoliberalism and the contamination of municipal water in Walkerton, Ontario. Geoforum.

[CR85] Qin B, Zhu G, Gao G (2010). A drinking water crisis in Lake Taihu, China: linkage to climatic variability and lake management. Environ Manage.

[CR86] Rakia R (2016) Black Lives Matters calls the Flint water crisis an act of “state violence.” Grist. https://grist.org/living/black-lives-matters-calls-the-flint-water-crisis-an-act-of-state-violence/. Accessed 25 Sep 2022

[CR87] Rammelt C, Masud Z, Boes J, Masud F (2014). Toxic injustice in the Bangladesh water sector: a social inequities perspective on arsenic contamination. Water Policy.

[CR88] Ranganathan M (2016). Thinking with Flint: racial liberalism and the roots of an American water tragedy. Capital Nat Social.

[CR89] Ravenscroft P, Brammer H, Richards K (2009). Arsenic pollution: a global synthesis.

[CR90] Sabatier P, Sabatier P (2007). The need for better theories. Theories of the policy process.

[CR91] Salvadori MI, Sontrop JM, Garg AX (2009). Factors that led to the Walkerton tragedy. Kidney Int.

[CR92] San Bernadino County Sun (2013) Hinkley: Roberta Walker’s research brought Erin Brockovich to town. In: Los Angeles Dly. News. https://www.dailynews.com/2013/07/09/hinkley-roberta-walkers-research-brought-erin-brockovich-to-town/. Accessed 30 May 2022

[CR93] Schwartz R, McConnell A (2009). Do crises help remedy regulatory failure? A comparative study of the Walkerton water and Jerusalem banquet hall disasters. Can Public Adm.

[CR94] Shamsudduha M, Joseph G, Haque SS (2020). Multi-hazard groundwater risks to water supply from shallow depths: challenges to achieving the sustainable development goals in Bangladesh. Expo Health.

[CR95] Smith AH (2008). Hexavalent chromium, yellow water, and cancer: a convoluted saga. Epidemiology.

[CR98] Smith M (2019). Flint water prosecutors drop criminal charges, with plans to keep investigating.

[CR96] Smith AH, Lingas EO, Rahman M (2000). Contamination of drinking-water by arsenic in Bangladesh: a public health emergency. Bull World Health Organ.

[CR97] Smith AH, Steinmaus CM (2009). Health effects of arsenic and chromium in drinking water: recent human findings. Annu Rev Public Health.

[CR99] Snider L (2004). Resisting neo-liberalism: the poisoned water disaster in Walkerton, Ontario. Soc Leg Stud.

[CR100] Statistics Canada (2019) 2001 Census: community highlights for Walkerton. https://www12.statcan.gc.ca/English/profil01/CP01/Details/Page.cfm?Lang=E&Geo1=CSD&Code1=3541036&Geo2=PR&Code2=35&Data=Count&SearchText=Walkerton&SearchType=Begins&SearchPR=01&B1=All&GeoLevel=PR&GeoCode=3541036. Accessed 31 Jan 2022

[CR101] Stefanakis AI, Becker JA (2016) A review of emerging contaminants in water: Classification, sources, and potential risks. In: McKeown A, Bugyi G (eds) Impact of water pollution on human health and environmental sustainability. pp 55–80. 10.4018/978-1-4666-9559-7.ch003

[CR102] Steinpress MG, Ward AC (2001). The scientific process and hollywood: the case of hexavalent chromium. Ground Water.

[CR104] Sultana F (2009). Fluid lives: subjectivities, gender and water in rural Bangladesh. Gend Place Cult.

[CR103] Sultana F (2011). Suffering for water, suffering from water: emotional geographies of resource access, control and conflict. Geoforum.

[CR105] Sutradhar v. Natural Environment Research Council (2006) UKHL 33

[CR106] Sutton R (2010) Chromium-6 in U.S. Tap Water. Environmental Working Group. http://www.annarbor.com/EWG-report.pdf. Accessed 25 Sep 2022

[CR107] The Economist Intelligence Unit (2021) Democracy Index 2020: In sickness and in health? https://www.eiu.com/n/campaigns/democracy-index-2020/. Accessed 25 Sep 2022

[CR108] The World Bank (2022) DataBank. https://databank.worldbank.org/home.aspx. Accessed 29 May 2022

[CR109] United Nations (1946). Convention on the privileges and immunities of the United Nations.

[CR110] US Census Bureau (2021) U.S. Census Bureau QuickFacts: Flint City, Michigan. https://www.census.gov/quickfacts/fact/table/flintcitymichigan/PST045219. Accessed 31 Jan 2022

[CR111] US EPA (2019) EPA’s proposed lead and copper rule revisions general fact sheet. https://www.epa.gov/ground-water-and-drinking-water/epas-proposed-lead-and-copper-rule-revisions-general-fact-sheet. Accessed 31 Jan 2022

[CR112] US EPA (2021) Chromium in drinking water. https://www.epa.gov/sdwa/chromium-drinking-water. Accessed 31 Jan 2022

[CR113] Waldman P (2005) Study tied pollutant to cancer; then consultants got hold of it. Wall Street Journal, New York. https://www.wsj.com/articles/SB113530126572230084. Accessed 25 Sep 2022

[CR116] WHO (2005) A field guide for detection, management and surveillance of arsenicosis cases. https://apps.who.int/iris/bitstream/handle/10665/204725/B0301.pdf?sequence=1&isAllowed=y. Accessed 31 Jan 2022

[CR114] WHO (2010). Childhood lead poisoning.

[CR115] WHO (2018) *Escherichia coli*. https://www.who.int/news-room/fact-sheets/detail/e-coli. Accessed 31 Jan 2022

[CR117] WHO (2021) Manganese in drinking-water: Background document for development of WHO Guidelines for drinking-water quality. https://apps.who.int/iris/bitstream/handle/10665/350933/WHO-HEP-ECH-WSH-2021.5-eng.pdf?sequence=1&isAllowed=y. Accessed 25 Sep 2022

[CR118] Yu WH, Harvey CM, Harvey CF (2003). Arsenic in groundwater in Bangladesh: a geostatistical and epidemiological framework for evaluating health effects and potential remedies. Water Resour Res.

[CR119] Zahariadis N, Sabatier P (2007). The mulitple streams framework. Theories of the policy process.

[CR120] Zahran S, McElmurry SP, Kilgore PE (2018). Assessment of the Legionnaires’ disease outbreak in Flint, Michigan. Proc Natl Acad Sci USA.

